# New Appliances & Things Medical

**Published:** 1910-09-24

**Authors:** 


### NEW APPLIANCES & THINGS MEDICAL.

**[We shall be glad to receive at our Office, 28 & 29 Southampton Street, Strand, London, W.C., from the manufacturers, specimens of all new preparations and appliances.]**

### SKEFFINGTON'S PATENT INVALID LIFTERS.

(SKEFFINGTON, 49 ULUNDI ROAD, BLACKHEATH, S.E.)

THESE useful and practical lifters, which are easily slipped on to any hospital bed by a few simple clamps, deserve to be more widely known than they are at present for they supply a decided and long-felt want. The lifter, which enables a child to raise a full-grown patient from the bed by a few simple turns of the handle, consists of a hardwood roller fixed to the head of the bedstead, to which is attached a special strong sheet by means of a steel rod passing through a hem in the end of the sheet and fitting into a groove in the roller. The roller is rotated by a worm gearing and handle which, when turned, draws both patient and sheet towards the head of the bed. The Anastasia pattern is a folding mobile and compact form of lifter, which is specially suitable for private and institutional use. These lifters, it is interesting to note, have been awarded the silver medal at the recent Royal Sanitary Institute's Health Exhibition held at Brighton during the present month. There were sixteen silver medals awarded in all at this exhibition, but no gold medals, and the honour awarded to the Skeffington Lifters is therefore a distinguished tribute to their merit.

